# Chest Tube Insertion in Prone Position Using Ultrasound in a COVID 19 Patient

**DOI:** 10.7759/cureus.27526

**Published:** 2022-07-31

**Authors:** Suhail Yaqoob Hakim, Tahir Shahzad

**Affiliations:** 1 Trauma Surgery, Hamad Medical Corporation, Doha, QAT; 2 Emergency Medicine, Hamad Medical Corporation, Doha, QAT

**Keywords:** left-sided pleural effusion, covid-19, chest tube, ultrasound-guided, prone positioning

## Abstract

Chest tube insertion is one of the most common interventions performed to manage pleural effusion, pneumothorax, haemothorax, etc. The procedure is done conventionally in a supine position, and a triangle of safety is used as a landmark. COVID-19 is a multiorgan disorder, declared a pandemic by WHO, predominantly involving the respiratory system. To insert a chest tube in a prone position using the Seldinger technique is quite a unique way of doing this common procedure with unclear complications in literature. COVID-19 is a highly infectious disease that makes chest tube insertion a risky procedure for operators as well due to direct exposure to respiratory secretions. Full personal protective equipment and a negative pressure room are used during this procedure in this case. The case mentions the ultrasound-guided chest tube insertion in a COVID-19 patient with severe acute respiratory distress syndrome (ARDS) and significant left-sided pleural effusion requiring prone positioning and mechanical ventilation.

## Introduction

Thoracostomy tube placement is one of the most common bedside interventions performed either emergently or electively based on its indication. The standard site to insert a thoracostomy tube is the fourth or fifth intercostal space in the mid or anterior axillary line [1]. The use of bedside ultrasound is tremendously increased and improved the safety of the procedure, helping in identifying the optimal sites and avoiding potential complications [2]. Prone positioning is advocated in severe acute respiratory distress syndrome (ARDS) to improve lung mechanics and ventilation-perfusion match, resulting in better oxygenation and ventilation [3]. The prone position is an atypical position to place a thoracostomy tube due to distorted anatomical landmarks and lack of literature related to technique and potential complications. Severe acute respiratory syndrome coronavirus 2 (SARS-Cov-2) is a viral pathogen responsible for severe coronavirus disease 2019 (COVID-19); a pandemic emerged in late January 2020 and has taken more than 6,311,088 lives until 16th June 2022 as per the World Health Organization (WHO) [4]. Patients can develop ARDS in severe COVID-19 infections requiring different life-saving measures, including prone positioning as well. In this case, a young male with a severe COVID-19 infection and ARDS was put in a prone position and found to have a significant left-sided pleural effusion, for which a thoracostomy tube was placed in the prone position using bedside ultrasound.

## Case presentation

**Figure 1:**
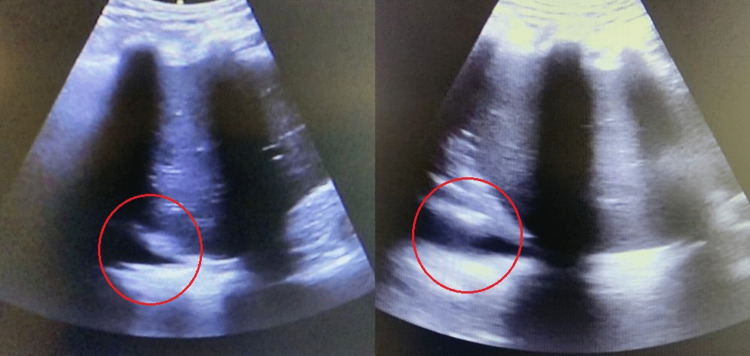
Bedside ultrasound Red circles highlight subdiaphragmatic pleural effusion

A 58-year-old male was admitted to the intensive care unit (ICU) due to worsening shortness of breath (SOB), decreasing oxygen saturation, and hemodynamic instability. He initially presented to the emergency department (ED) with 10 days of dry cough associated with fever and SOB for the last two days. In the ICU, he was intubated and mechanically ventilated which resulted in some improvement in his clinical condition. On the third day of his ICU admission, his vital signs were noted to be a heart rate of 118 beats/minute, blood pressure of 110/43 mmHg, and oxygen saturation was 90% on 100% FiO2 (continuous mandatory ventilation (CMV) mode - positive end-expiratory pressure (PEEP) 8). The patient was put in a prone position and observed; however, this did not improve the condition. A bedside ultrasound was done, which showed significant left-sided pleural effusion (Figure 1). The decision was made to insert a thoracostomy tube in the prone position via the Seldinger technique under ultrasound guidance. A large collection of pleural fluid was identified using bedside ultrasound, and this was marked on the patient’s skin as the site of insertion. After all aseptic measures were taken, and full personal protective equipment was donned, a needle was inserted under ultrasound (US) guidance at the marked site, inferior and lateral to the inferior angle of the left scapula, next to the triangle of safety’s lateral border (latissimus dorsi muscle), almost touching the mid-axillary line.

**Figure 2:**
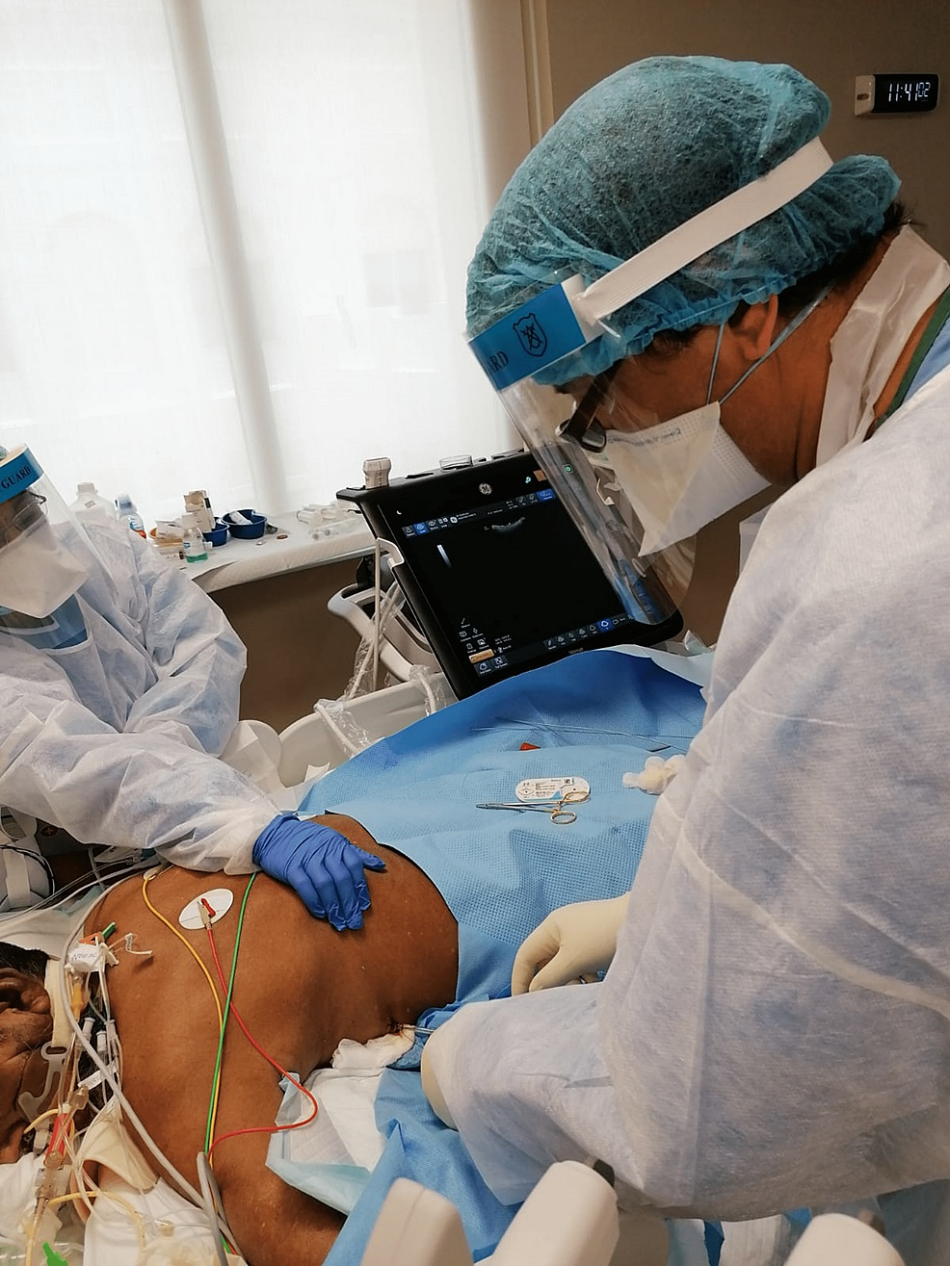
Securing thoracostomy tube

The needle’s direction was anteroinferior, and the needle was seen in line with the US. Aspiration was performed with a return of light yellowish-colored fluid. A guide wire was then introduced through the needle, and the thoracostomy tube was placed using the Seldinger technique. The procedure was completed without any immediate or delayed obvious adverse effect (Figure 2).

**Figure 3:**
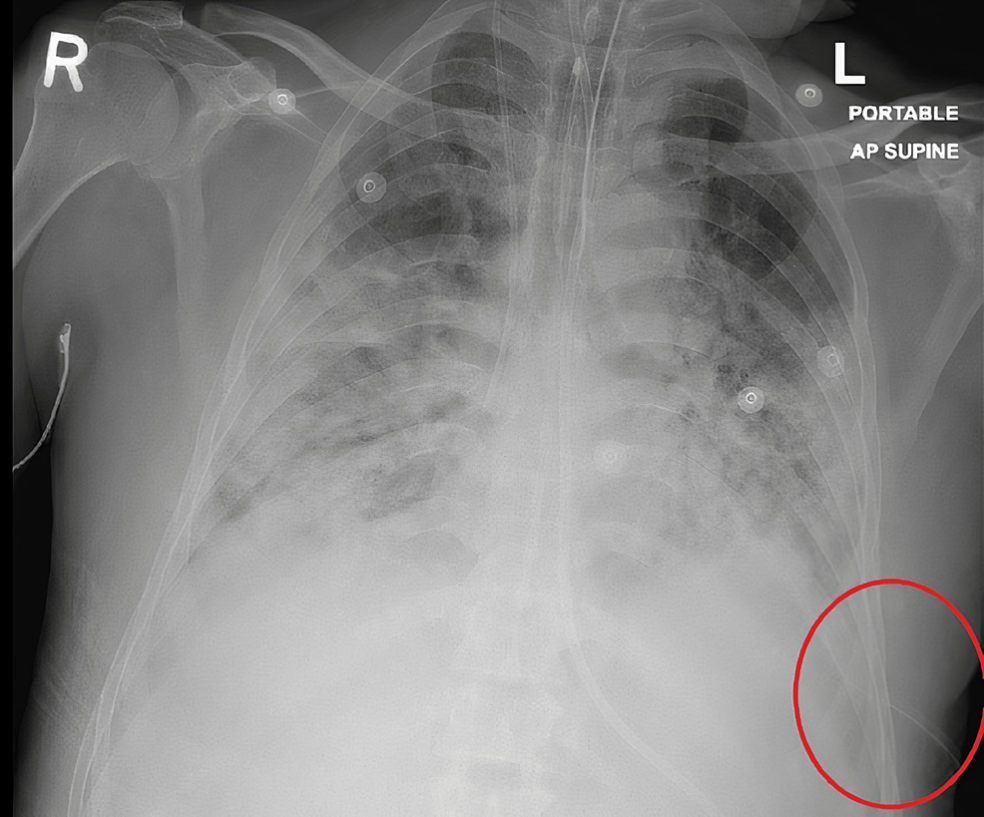
Chest X-ray Post thoracostomy tube insertion

The chest X-ray (Figure 3) was done the next day after the patient was put in a supine position, showing a thoracostomy tube in place on the left side. Thoracostomy tube insertion improved oxygenation and respiratory distress.

The patient developed multi-organ failure, requiring hemodialysis and persistent vasopressor support as well due to shock. He eventually suffered from cardiac arrest and died on the 28th day of his admission to the hospital.

## Discussion

Prone positioning in ARDS helps recruit dorsal-dependant atelectatic alveoli resulting in improved gas exchange and oxygenation; it also reduces hyperinflation of the non-dependant alveoli [5]. In severe COVID-19 infections complicated by ARDS, prone positioning is of proven benefit [6]. Pleural effusion is a rare finding in COVID-19 infection [7]; however, it should be considered as a potential contributor to clinical instability, as in this case as. Thoracostomy tube placement is associated with either technical or infectious complications. Common technical complications include malposition, dislodgement, blocked drain, nerve injuries, and cardiac and vascular injuries. Infectious complications include empyema and surgical site infections [8]. The mentioned complications are reported for a normal supine position; however, when it comes to prone positioning, the possible complications can be more due to technical reasons related to its non-conventional approach, less operator's experience, and lack of recommended landmarks (triangle of safety) [9]. In the prone position, the technique of placing a thoracostomy tube is not well described, which can be due to its rare need before COVID 19 pandemic; however, one of the articles mentions putting a rolled blanket under the lateral chest to expose the triangle of safety and aim the midaxillary line [10]. In this case, a different approach is adopted, resulting in the successful placement of a thoracostomy tube in a prone position to drain pleural effusion without obvious complications. The use of bedside ultrasound is becoming more common due to its portability and safety profile; its role is equally important for the assessment and management of pleural effusion in critically ill patients [11]. Bedside ultrasound played a critical role in this case, helping to perform tube thoracostomy in a prone position. It helps to identify the optimal site and real-time visualization of the needle [12], making placement of the thoracostomy tube safer and more successful.

## Conclusions

Thoracostomy tube placement in a prone position is not well described or researched, specifically in COVID-19 patients. The potential complications related to it can be more due to technical challenges and uncommon landmarks. The use of bedside ultrasound is of utmost importance and must be regularly used to avoid any harm and optimize procedural outcomes. 

## References

[1] Huggins JT, Carr SR, Woodward GA (2022). Thoracostomy tubes and catheters: placement techniques and complications. UpToDate.

[2] Daniels CE, Ryu JH (2011). Improving the safety of thoracentesis. Curr Opin Pulm Med.

[3] Pelosi P, Tubiolo D, Mascheroni D, Vicardi P, Crotti S, Valenza F, Gattinoni L (1998). Effects of the prone position on respiratory mechanics and gas exchange during acute lung injury. Am J Respir Crit Care Med.

[4] (2022). WHO coronavirus (COVID-19) dashboard. https://covid19.who.int/.

[5] Sottile PD, Albert RK, Moss M (2022). Prone positioning for nonintubated patients with COVID-19-potential dangers of extrapolation and intermediate outcome variables. JAMA Intern Med.

[6] Langer T, Brioni M, Guzzardella A (2021). Prone position in intubated, mechanically ventilated patients with COVID-19: a multi-centric study of more than 1000 patients. Crit Care.

[7] Hussein MS, Ul Haq I, Thomas M, Allangawi M, Elarabi A, Hameed M (2020). Pleural effusion secondary to COVID-19 infection. Chest.

[8] Kesieme EB, Dongo A, Ezemba N, Irekpita E, Jebbin N, Kesieme C (2012). Tube thoracostomy: complications and its management. Pulm Med.

[9] Laws D, Neville E, Duffy J (2003). BTS guidelines for the insertion of a chest drain. Thorax.

[10] Dhanasopon AP, Zurich H, Preda A (2021). Chest tube drainage in the age of COVID-19. Physician Assist Clin.

[11] Bouhemad B, Zhang M, Lu Q, Rouby JJ (2007). Clinical review: Bedside lung ultrasound in critical care practice. Crit Care.

[12] Eisen L Chest tube insertion. Critical Care.

